# P-1485. Comparison of Eravacycline and Cefiderocol in the Treatment of Multidrug-resistant *Acinetobacter baumannii* Infections

**DOI:** 10.1093/ofid/ofae631.1655

**Published:** 2025-01-29

**Authors:** Justin Halim, Madeline King, Dejan Nikolic, Carlo Foppiano Palacios

**Affiliations:** Cooper Medical School of Rowan University, Camden, New Jersey; Cooper University Hospital, Camden, New Jersey; Cooper University Health Care, Camden, New Jersey; Cooper University Health Care, Camden, New Jersey

## Abstract

**Background:**

*A. baumannii* is a common cause of drug-resistant hospital-associated infections. Eravacycline and cefiderocol were recently introduced as agents against drug-resistant *A. baumannii*. Data comparing these drugs against multi-drug resistant (MDR) *A. baumannii* is lacking. We compared clinical outcomes of patients with multi-drug resistant *A. baumannii* infections treated with either eravacycline or cefiderocol.

**Methods:**

A retrospective chart review of patients with MDR *A. baumannii* infections from 2019-2023 at Cooper University Hospital who received either eravacycline or cefiderocol was performed. MDR infections were defined as isolates resistant or intermediate to meropenem or ampicillin-sulbactam. Patients were excluded if *A. baumannii* was deemed a colonizer rather than infection per infectious diseases evaluation, or non-MDR infections. We collected data on demographics, risk factors for MDR infection, and infection characteristics. Primary outcome was hospital mortality rate. Secondary outcomes assessed were mortality and readmission at 30 and 90 days, ICU admission, shock requiring vasopressors, mechanical intubation, and drug-related adverse events. Descriptive statistics and Fisher’s exact test were used.

**Results:**

44 patients were included, median age was 61 years old, 80% were male, 59% identified as White, and 13.6% were Latino. Most common site of infection was pulmonary (59%). 31 patients received cefiderocol, 11 received eravacycline, and 2 received combination therapy with both agents. Higher hospital mortality rates were observed in the cefiderocol group (52% vs 18%), approaching significance (P=0.08). Hospital mortality, and 30 and 90-day mortality were not significant between both groups. Patients on cefiderocol were more likely to require mechanical intubation (74% vs 27%, P=0.01). ICU admission and use of vasopressors did not differ significantly between both groups. Liver function elevations more common among patients on cefiderocol compared to eravacycline (61% vs 9%, P=0.004).

**Conclusion:**

In this retrospective study comparing eravacycline and cefiderocol for the treatment of MDR *A. baumannii* infections, mortality and readmission outcomes were comparable between both groups.

**Disclosures:**

**Madeline King, PharmD**, Innoviva: Advisor/Consultant

